# Toward LLM-aware software effort estimation: a conceptual framework

**DOI:** 10.3389/frai.2026.1772418

**Published:** 2026-03-23

**Authors:** Feisal Alaswad, Eswaran Poovammal, Batoul Aljaddouh

**Affiliations:** Department of Computing Technologies, SRM Institute of Science and Technology, Kattankulathur, Tamil Nadu, India

**Keywords:** AI-augmented software engineering, software effort estimation, large language models (LLMs), human-AI collaboration, AI-assisted software development, agile user stories, hybrid intelligence

## Abstract

Software effort estimation has traditionally been grounded in the assumption that development cost is primarily driven by human labor, approximated through proxies such as code size, functional complexity, or perceived task difficulty. The increasing adoption of large language models (LLMs) as software development assistants challenges this assumption by automating substantial portions of reasoning, coding, and refactoring. In LLM-assisted workflows, effort increasingly shifts toward interaction management, validation, correction, and integration, leading to growing misalignment between established estimation techniques—such as COCOMO, Function Points, and Story Points—and actual development cost. This paper argues that the limitations of existing estimation models in LLM-mediated development are structural rather than parametric. When core development activities are delegated to automated reasoning systems. Through conceptual analysis supported by exploratory observations, we illustrate systematic mismatches between traditional effort estimates and LLM-assisted task execution, particularly in agile environments that rely on Story Points. To address this gap, we introduce a unified conceptual foundation for LLM-aware software effort estimation. We reconceptualize effort as *Hybrid Intelligence Effort*, emerging from the interaction between LLM cognitive complexity and human oversight effort. We further identify five core dimensions governing effort in LLM-assisted development: LLM reasoning complexity, context and information completeness, code transformation impact, iterative reasoning cycles, and human oversight effort. These dimensions capture cost drivers that are largely absent from conventional estimation theory. Rather than proposing a parametric estimation model, this work establishes a theoretical foundation for future empirical calibration and data-driven approaches. By redefining what constitutes effort in the presence of LLMs, the paper contributes a conceptual basis for estimation models aligned with contemporary AI-augmented software engineering practices.

## 1 Introduction

Software effort estimation has been a foundational activity in software engineering for decades, serving as a critical input for project planning, resource allocation, budgeting, scheduling, and risk management (Sharma and Singh, 2017). A wide range of estimation techniques—spanning algorithmic cost models (e.g., COCOMO), functional sizing approaches (e.g., Function Points), expert-based methods, data-driven machine-learning techniques, and agile practices such as Story Points—have been proposed and refined to support these activities. Despite their methodological differences, these approaches share a common underlying assumption: software development effort is primarily driven by human labor (Rastogi et al., 2014; Cohn, 2005). Effort is therefore approximated indirectly through proxies such as source code size, functional complexity, system interfaces, or the perceived cognitive difficulty of tasks. This assumption, while historically valid, is becoming increasingly fragile.

The rapid adoption of large language models (LLMs) as software development assistants has fundamentally altered how software is produced (Peng et al., 2023). Contemporary LLMs are now capable of generating substantial amounts of syntactically correct and semantically meaningful code (Wang and Chen, 2023), implementing well-known algorithms (Talebirad et al., 2025), producing unit and integration tests (Yang et al., 2024), refactoring existing codebases (Midolo and Tramontana, 2025), and translating high-level requirements into executable/design artifacts (Alaswad et al., 2023). Tasks that previously required hours or days of manual coding can now often be completed within minutes through iterative prompting and minor human adjustments (Huynh and Lin, 2025).

In this emerging development paradigm, traditional proxies for effort—such as lines of code, number of functional components, or perceived task difficulty—no longer exhibit a stable or predictable relationship with actual development cost. LLMs can generate large volumes of code with minimal effort. In contrast, even small changes often trigger extensive validation, correction, and human oversight cycles (Tang et al., 2024). As a result, effort increasingly shifts away from manual code production toward activities such as managing LLM reasoning behavior, providing sufficient contextual information, reviewing generated artifacts, validating correctness, and mitigating hallucinations or incorrect assumptions.

These shifts introduce a growing disconnect between established estimation practices and actual development realities. In an exploratory empirical evaluation, we identified systematic misalignments between traditional human-centric estimation techniques and LLM-assisted task execution. Specifically, tasks labeled as highly complex in historical datasets (e.g., Neo Dataset Neo et al., 2024) were frequently completed with minimal LLM-assisted effort, whereas tasks classified as moderate or simple often incurred disproportionately high costs due to ambiguity, legacy constraints, or integration challenges requiring extensive iteration and human oversight. Quantitatively, in 78% of tasks labeled high-complexity, LLMs completed the work using less than 25% of the expected human effort, while approximately 22% of low-complexity tasks demanded over 180% of the anticipated effort due to validation and integration overhead. Table 1 summarizes a small set of illustrative observations drawn from an exploratory analysis of LLM-assisted development tasks, with methodological details reported in Supplementary material. These observations are intended to highlight recurring patterns of misalignment between traditional human-centric effort estimates and observed effort in LLM-mediated workflows, rather than to provide predictive validation or support statistical generalization.

**Table 1:** Illustrative exploratory observations highlighting potential misalignment between traditional effort estimates and LLM-assisted development effort.

**Traditional estimation category**	**Observed LLM-assisted effort pattern**	**Illustrative observation**	**Tentative conceptual implication**
High-complexity tasks	Substantially reduced execution effort	78% of tasks historically labeled as high-complexity were completed using less than 25% of the expected human effort when supported by LLMs	Functional or perceived complexity may no longer reliably predict effort when reasoning and code generation are assisted by LLMs
Low-to-moderate complexity tasks	Disproportionately high oversight effort	Approximately 22% of tasks labeled as low-complexity required more than 180% of the anticipated effort due to validation, integration, and correction overhead	Human oversight and verification effort could dominate total cost even for small-scope tasks
Tasks aligned with common patterns	Minimal interaction and validation cycles	Well-known algorithms and standard architectural patterns required few prompt–response iterations and limited human intervention	Effort may depend more on alignment with the LLM's learned representations than on artifact size
Tasks involving legacy systems or implicit constraints	Extensive iteration and supervision	Small localized changes frequently triggered cascading validation cycles due to undocumented assumptions and integration dependencies	Effort appears to shift from construction toward risk mitigation and system-level reasoning in such cases
Tasks with ambiguous or incomplete requirements	High variability and non-linear effort	Repeated prompt refinement and clarification cycles were required despite limited functional scope	Iterative reasoning dynamics, rather than scope alone, may become a primary driver of effort

Consistent with the illustrative observations, studies show that while LLMs substantially lower coding effort for well-defined tasks, developer effort is often still dominated by iterative reasoning, validation, and debugging, indicating that task scope or complexity alone cannot reliably predict effort in LLM-assisted workflows (Wang et al., 2024; Vaithilingam et al., 2022). This misalignment becomes especially pronounced in agile environments that rely on Story Points as the primary estimation mechanism. Story Points are designed to capture human-perceived difficulty and uncertainty (Khan et al., 2025), but these dimensions do not align with the dominant cost drivers in LLM-mediated development, such as prompt refinement, constraint handling, and integration overhead. As a result, Story Points become increasingly unstable and context-dependent in LLM-assisted workflows, revealing a growing mismatch between what they are designed to capture—human-perceived difficulty—and the dominant cost drivers of LLM-mediated development. While this paper discusses multiple estimation paradigms that traditionally assume human effort as the primary driver of code construction and task completion, Story Points are highlighted here as a representative case due to their prevalence in agile environments and their role in capturing overall work effort—including complexity, uncertainty, and risks— across user stories and backlog items in planning and sprint estimation practices, making them broadly applicable even when compared with other task-level estimation techniques (Peter et al., 2026).

Importantly, we do not attribute this mismatch solely to calibration or inexperience with LLMs. Instead, our results suggest a conceptual shift: traditional estimation models assume human cognitive effort is the dominant cost driver, whereas LLM-assisted production shifts effort toward supervision, validation, and integration. Estimators that ignore this shift may systematically mispredict costs.

This paper argues that software effort estimation must be fundamentally redefined for LLM-driven development. Rather than estimating effort solely as a function of human coding activity, we conceptualize it as a combination of *LLM cognitive complexity* and *human oversight effort*: the former captures how challenging a task is for an LLM to reason about and execute correctly, while the latter reflects the human effort required to validate, correct, and integrate LLM-generated artifacts. Our goal is not to introduce another parametric estimation model, but to provide a conceptual foundation for understanding effort in modern software engineering. By re-examining what “effort” means in the presence of LLMs, we clarify why existing theories are increasingly inadequate, identify the dimensions that now dominate development cost, and propose a unified framework that aligns estimation practices with contemporary development realities.

## 2 Background and related works

Software effort estimation has been studied extensively across multiple paradigms, including expert-based judgment, algorithmic cost models (Boehm et al., 1995), functional sizing techniques, agile estimation practices, and data-driven machine-learning approaches (Nalluri et al., 2023). Early work focused on statistical and model-based strategies such as COCOMO, Function Points, and related algorithmic models that approximate development effort through size- or complexity-oriented proxies (Sharma and Singh, 2017; Rastogi et al., 2014). Agile methods later introduced relative estimation mechanisms, most notably Story Points, to capture perceived difficulty, uncertainty, and risk in iterative development settings (Cohn, 2005; Khan et al., 2025). Despite methodological differences, these approaches share a common focus on estimating human development effort based on historical project data.

Subsequent research explored data-driven and machine-learning-based estimation techniques, leveraging structured project artifacts and historical records to predict effort-related outcomes such as person-hours or Story Points. Prior studies report improvements in predictive accuracy when sufficient labeled data are available (Ahmad and Kuo, 2025). However, existing work also highlights persistent challenges related to model explainability, data sparsity, and practical integration into planning workflows, limiting the broader adoption of such approaches in industrial contexts.

With the emergence of LLMs, recent studies have begun examining their role in effort estimation and planning activities. Existing work primarily investigates the use of LLMs to support estimation tasks, such as predicting Story Points, assisting backlog refinement, or automating parts of planning processes (Carpenter et al., 2024; Pavlič et al., 2024; Bairi et al., 2024; Coelho Jr et al., 2024). Systematic mapping studies report rapid growth in this research area, identifying increased interest in generative AI for early-stage estimation while also noting gaps in methodological rigor and contextual grounding (Radliński and Swacha, 2025).

Several framework-level contributions propose multi-agent or hybrid human–AI approaches to estimation, emphasizing coordination between automated suggestions and human judgment (Bui et al., 2025). Complementary empirical and survey-based studies document how LLM adoption reshapes development practices, showing that while code generation effort may decrease, developers remain heavily involved in reviewing, validating, debugging, and integrating generated artifacts (Santos et al., 2025). Prior work also reports a productivity–quality trade-off, where gains in generation speed are offset by increased effort in evaluation and correction (Tabarsi et al., 2025).

Overall, existing literature demonstrates growing interest in AI-assisted estimation and highlights changing development practices in the presence of LLMs. However, most prior work treats LLMs primarily as tools for improving estimation accuracy or productivity within established human-centric estimation frameworks. As a result, while recent studies capture emerging patterns of human–AI collaboration, they do not explicitly reconsider the underlying definition of software effort in settings where reasoning, construction, and validation are partially delegated to automated systems. This limitation motivates the present work, which focuses on redefining the estimation target itself rather than proposing another predictive technique.

## 3 Revisiting the assumptions of software effort estimation

### 3.1 Why traditional estimation models break down in LLM-driven development

#### 3.1.1 Collapse of size-based proxies

Traditional software effort estimation models, such as COCOMO and Function Point Analysis, rely on proxies like lines of code, numbers of inputs and outputs, files, and system interfaces to predict development effort. In workflows augmented by LLMs, these proxies lose much of their explanatory power. LLMs are capable of producing large volumes of syntactically correct code in seconds, rendering code size and incremental development assumptions poor predictors of effort. While models such as COCOMO II can, in principle, account for complexity, reliability, and other quality drivers, the application of such models to LLM-assisted development remains inherently subjective. Key factors in LLM-driven workflows—such as prompt engineering complexity, reasoning depth, iterative generation cycles, code validation, and system integration—cannot yet be parameterized, quantified, or scaled in a manner compatible with traditional numeric effort drivers. As a result, conventional models struggle to provide accurate or meaningful effort estimates in LLM-assisted development contexts.

#### 3.1.2 Instability of story points

Story Points rely on developers' prior experience and perceived difficulty to gauge task effort (Khan et al., 2025). While familiar tasks are often assigned lower estimates and unfamiliar tasks higher ones, these judgments reflect human-centric cognitive and experiential factors. In contrast, effort in LLM-assisted development is shaped by a different set of determinants that are largely absent from Story Point estimation, including prompt engineering complexity, validation and oversight overhead, integration risk, and the degree of alignment between task structure and the model's learned representations. Consequently, Story Point estimates become unstable in LLM-assisted workflows not because task familiarity is uniformly reduced, but because Story Points are insensitive to these LLM-specific cost drivers. As a result, tasks with similar Story Points may exhibit markedly different effort profiles when executed with LLM assistance.

#### 3.1.3 Structural mismatch: the conceptual obsolescence of generative metrics

The inadequacy of COCOMO II in estimating AI-augmented development is fundamentally *structural* rather than *parametric*. Traditional software cost estimation models are calibrated on the foundational assumption that human effort is the primary engine of both reasoning and construction. Consequently, no degree of statistical recalibration can bridge this gap without redefining the estimation target itself to account for the shift from human construction to delegated reasoning and verification-centric oversight.

In the COCOMO II framework, the exponent *E* represents the diseconomy of scale, as expressed in the core effort Equation 1.


$$\begin{array}{l} Effort=A\times {\left(Size\right)}^{E}\times \overset{n}{\underset{i=1}{\prod }}EM_{i} \end{array}$$ (1)

Standard models assume that as project size increases, complexity grows exponentially (*E* > 1.0) due to communication overhead and integration friction. However, in AI-delegated workflows, the *Construction* phase becomes nearly instantaneous and decoupled from human effort.

The main challenge shifts from writing code to verifying how different parts of the system behave together. Because current LLMs do not maintain a complete, system-wide understanding of large codebases, they may introduce changes that appear correct locally but cause failures elsewhere. As a result, human effort is concentrated on tracing, testing, and validating these interactions, and this verification work can grow more rapidly with system size than traditional manual coding effort.

A primary conceptual tension lies in the treatment of iteration. COCOMO II traditionally categorizes rework as a waste metric, typically stemming from volatile requirements or poor architectural planning. In the AI paradigm, this logic is inverted.

In these workflows, the *cycle of refinement* (prompting, testing, and adjusting) is the primary unit of productive work. The model must be conceptually refactored to treat these iterative loops not as signs of failure, but as the fundamental mechanism of construction. The human role has evolved from a builder to a curator and risk-mitigator, rendering Source Lines of Code (SLOC)-based productivity metrics obsolete in favor of verification density and reasoning-audit hours.

### 3.2 From human effort to hybrid intelligence effort

LLM-driven software development necessitates a redefinition of what constitutes *effort*. Rather than viewing effort as exclusively human labor, we introduce the notion of *Hybrid Intelligence Effort*, defined as the combined cost of machine-performed reasoning and human-performed supervision required to produce correct, deployable software artifacts.

In this paradigm, LLMs assume substantial portions of the cognitive workload traditionally attributed to developers, including requirement interpretation, algorithm synthesis, and code generation. Human effort is correspondingly reallocated toward activities that govern and validate delegated reasoning, such as contextual grounding, prompt refinement, output inspection, test design, failure diagnosis, and system integration.

As reasoning, construction, and validation are no longer tightly coupled human activities, software effort can no longer be modeled as a monotonic function of artifact size, functional scope, or perceived task difficulty. Instead, effort emerges from interaction dynamics between human supervisors and machine-generated outputs, where cost is driven primarily by verification intensity, correction cycles, ambiguity resolution, and integration risk rather than construction alone.

Crucially, Hybrid Intelligence Effort is not reducible to static properties of the software artifact. Two tasks with identical functional scope may incur radically different effort depending on their alignment with the LLM's internal representations, the clarity of requirements, and the degree of oversight required to ensure correctness. This interaction-driven perspective establishes a theoretical foundation for rethinking software effort estimation in LLM-mediated development.

## 4 Conceptual foundations of LLM-aware software effort estimation

In LLM-assisted development, substantial portions of implementation are delegated to automated reasoning systems, shifting effort away from manual coding toward interaction, supervision, and validation. As a result, software effort is governed by a set of dimensions that are largely absent from conventional estimation frameworks. The term “LLM effort” does not imply economic cost or labor expenditure by the model; rather, it denotes the cognitive complexity of tasks delegated to the LLM and the resulting interaction and oversight effort required from human developers.

**Figure 1:**
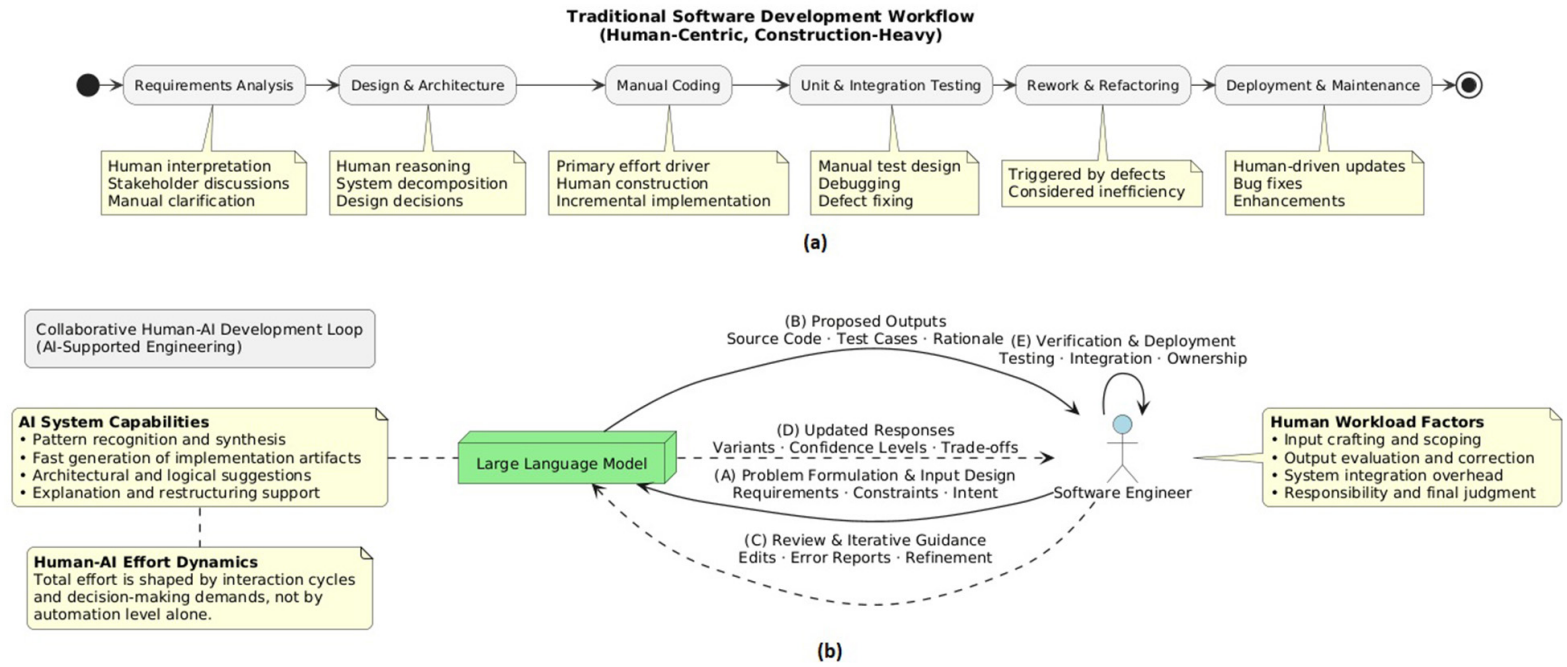
Comparison of development workflows: **(a)** Traditional human-centric software development workflow and **(b)** Collaborative human–AI development loop.

Figure 1 contrast traditional human-centric development workflows with contemporary LLM-assisted workflows. In the traditional model (Figure 1a), effort is dominated by linear, construction-heavy phases in which human reasoning, manual coding, and incremental implementation constitute the primary cost drivers. Rework and refactoring are typically treated as inefficiencies triggered by defects or requirement volatility.

In contrast, the LLM-assisted workflow (Figure 1b) is inherently iterative and interaction-driven. Core construction activities are partially delegated to the LLM, while human effort shifts toward problem formulation, constraint specification, review, verification, and integration. Productive work emerges through repeated prompt–response–refinement cycles rather than linear progression through predefined phases. As a result, effort is no longer proportional to code volume or functional size, but instead arises from interaction dynamics, reasoning alignment, and oversight intensity. This contrast illustrates why traditional estimation models, which assume effort is primarily driven by human construction activities, exhibit a structural mismatch when applied to LLM-mediated development.

Based on conceptual analysis, we identify five core dimensions that collectively characterize effort in LLM-driven software development workflows:

- LLM Reasoning Complexity. This dimension characterizes the emergent interaction difficulty observed when delegating a task to a LLMs, as inferred from observable interaction dynamics—not from assumptions about the model's internal cognitive states. It reflects the misalignment between task requirements and the model's learned representations and reasoning behaviors, manifested through metrics such as error rates, response inconsistency, uncertainty in outputs, and the frequency of required refinements. Tasks requiring multi-step reasoning, constraint handling, architectural decisions, or cross-module integration typically induce higher LLM reasoning complexity, as they demand prolonged interaction and supervision to achieve stable, correct solutions.
LLM Reasoning Complexity = The emergent difficulty ofaligning task requirements with the model's reasoning behavior,observable through interaction patterns
- Context and Information Completeness. This dimension reflects the degree to which all necessary information is explicitly available to the LLM at prompt time. Tasks with complete and well-scoped context can often be solved efficiently, whereas incomplete, ambiguous, or distributed information requires the LLM to infer missing details across multiple prompts, increasing interaction effort and the risk of misalignment.
$$\begin{array}{c} Context\;and\;Information\;Completeness\;=\;The\;extent\;to\;which\\ all\;task-relevant\;\inf ormation\;is\;\exp licitly\;available\;to\;the\;LLM\\ at\;prompt\;time \end{array}$$
- Code Transformation Impact. Code transformation impact refers to the scope and propagation of changes induced by a task, particularly as amplified by LLM-assisted generation. While localized modifications confined to a single file or function typically incur low effort, changes that propagate across multiple files, dependencies, or interfaces introduce elevated integration and regression risk. In LLM-assisted workflows, this risk is exacerbated by the model's limited ability to maintain a global, intent-aware understanding of large codebases, increasing the likelihood of non-local side effects that require extensive human validation and correction. As a result, transformation impact becomes a dominant driver of oversight effort even when direct code generation is inexpensive.
Code Transformation Impact = The extent to which a taskinduces changes that propagate across files, dependencies, andsystem components
- Iterative Reasoning Cycles. Unlike traditional coding, LLM-assisted development frequently proceeds through multiple prompt–response–refinement loops. This dimension captures the number of such cycles required to reach acceptable output quality. Tasks requiring frequent clarification, correction, or re-prompting incur higher effort despite minimal direct manual coding.
Iterative Reasoning Cycles = The number ofprompt–response–refinement loops required to achieveacceptable output quality
- Human Oversight Effort. Human oversight encompasses activities such as reviewing LLM outputs, validating correctness, writing and executing tests, correcting errors, managing edge cases, and mitigating risks related to security, performance, or maintainability. While LLMs can substantially reduce direct coding time, they introduce new forms of effort associated with supervision, validation, and accountability.
$$\begin{array}{c} Human\;Oversight\;Effort\;=\;The\;amount\;of\;human\;review,\\ validation,\;correction,\;and\;accountability\;required\;to\;ensure\\ the\;correctness\;and\;reliability\;of\;LLM-generated\;outputs \end{array}$$

While the identified dimensions are interrelated, they serve analytically distinct roles within the framework. Context and information completeness describes the initial availability and distribution of task-relevant information provided to the model. Iterative reasoning cycles represent the observable interaction outcome that emerges during task execution, reflecting the number of refinement loops required to reach acceptable output quality. LLM reasoning complexity captures the latent difficulty of aligning task requirements with the model's reasoning behavior, as revealed through instability, inconsistency, or repeated correction. Distinguishing these dimensions allows the framework to separate task conditions, interaction dynamics, and underlying sources of effort, rather than collapsing them into a single explanatory factor.

A central insight emerging from these dimensions is that LLM effort and human effort are complementary rather than interchangeable. Reductions in direct human construction effort are frequently offset by increases in oversight, validation, and coordination activities. Consequently, software effort in LLM-assisted development arises from the interaction between automated reasoning and human supervision, rather than from either component in isolation.

Importantly, LLM reasoning complexity is inferred from interaction dynamics and observable outputs, rather than from assumptions about internal cognitive states or intrinsic model effort.

**Figure 2:**
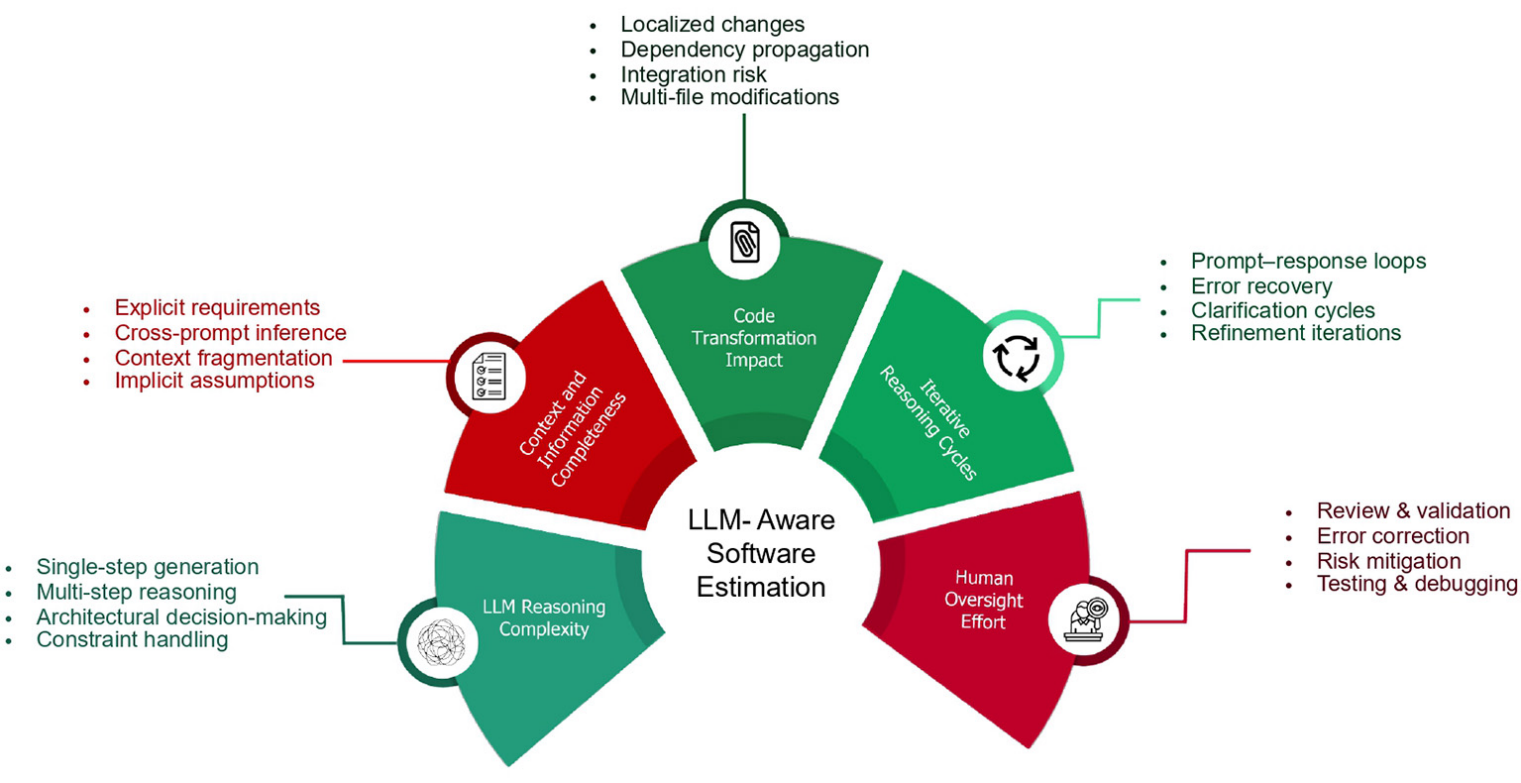
Conceptual dimensions governing software effort in LLM-assisted development. Effort emerges from the interaction between LLM cognitive complexity and human oversight, shaped by reasoning depth, context completeness, transformation scope, iterative cycles, and validation activities.

Building on these observations, and as illustrated in Figure 2, we conceptualize LLM-aware software effort as emerging within a two-dimensional space defined by LLM Cognitive Complexity and Human Oversight Effort. The former subsumes dimensions related to reasoning depth, context completeness, transformation impact, and iterative reasoning cycles, while the latter captures the residual human responsibility required to ensure correctness, reliability, and risk control. At this stage, these dimensions are intentionally not operationalized as numeric variables. Instead, they delineate the conceptual structure within which effort manifests in LLM-mediated development.

The definition of measurement scales, normalization strategies, and the relative weighting of individual dimensions are therefore deferred to future empirical investigation. Such weights cannot be assumed a priori, as their influence is likely to vary across domains, task types, development contexts, and levels of automation. Consequently, any aggregation of these dimensions into a unified effort estimate must be learned empirically from real-world datasets capturing LLM-assisted development activities. These datasets should reflect not only development outcomes, but also interaction dynamics such as prompt iterations, validation cycles, correction effort, and integration overhead. Accordingly, the present work establishes the theoretical foundation necessary for future data-driven estimation approaches, rather than proposing a prematurely parameterized effort equation.

## 5 Framework-grounded conceptual examples

This section provides analytically grounded examples that demonstrate how the proposed LLM-aware effort framework can be used to reason about development cost in LLM-assisted workflows. The examples are purely conceptual and are intended to illustrate how different configurations of the framework's dimensions—LLM cognitive complexity and human oversight effort—give rise to distinct effort profiles, even when traditional indicators such as functional scope or code volume suggest otherwise.

### 5.1 Illustrative example 1: high functional complexity, low hybrid effort

Consider a development task involving the implementation of a well-known algorithmic component, such as a role-based access control (RBAC) module with standard authorization rules. From a traditional estimation perspective, this task may be labeled as highly complex due to the number of roles, permissions, and interaction paths involved. However, in an LLM-assisted workflow, the task aligns closely with widely represented patterns in the model's training data. As a result, the LLM can generate a correct and complete implementation with minimal prompting and limited human intervention. Validation primarily consists of checking conformance to known specifications and executing standard test cases. In this scenario, both LLM cognitive complexity and human oversight effort remain low despite the task's apparent functional complexity, illustrating the collapse of traditional size- and complexity-based effort proxies. Within the proposed framework, this task occupies a region characterized by low LLM reasoning complexity and low human oversight effort, despite high functional complexity, illustrating why traditional complexity-based estimation fails in LLM-assisted development.

### 5.2 Illustrative example 2: limited functional scope, high oversight effort

Consider a development task characterized by limited functional scope, such as modifying the behavior of a configuration module within a mature legacy system. Although the required change affects only a small portion of the codebase, the surrounding system may embody undocumented assumptions, tight coupling, and historically accumulated workarounds.

In an LLM-assisted workflow, the model may generate a solution that appears locally correct yet inadvertently violates implicit system constraints or introduces regressions in distant components. Identifying and resolving these issues requires extensive human validation, iterative prompting, and manual reasoning about system-wide interactions. In this scenario, LLM cognitive complexity remains relatively modest, while human oversight effort dominates the overall cost.

Within the proposed framework, this task occupies a region characterized by low-to-moderate LLM reasoning complexity and high human oversight effort, demonstrating how effort can be driven primarily by verification and integration demands rather than by functional scope or code volume.

### 5.3 Illustrative example 3: ambiguous requirements and iterative reasoning loops

As a third example, consider a task defined by loosely specified or evolving requirements, such as implementing a business rule derived from informal stakeholder discussions. For human developers, such tasks are often resolved through clarification meetings and incremental refinement. In an LLM-assisted workflow, ambiguity manifests as repeated cycles of prompt refinement, partial generation, validation failures, and corrective feedback. Each iteration incurs additional human effort, even when the generated code is syntactically correct. Here, effort emerges from sustained interaction dynamics rather than from construction alone. This example illustrates how LLM cognitive complexity—driven by requirement ambiguity—interacts with human oversight effort to produce non-linear and difficult-to-predict development costs. In framework terms, ambiguous requirements increase LLM reasoning complexity and trigger repeated iterative reasoning cycles, which in turn amplify human oversight effort, producing non-linear effort growth unrelated to code size.

These examples are illustrative in nature and are intended solely to demonstrate how the proposed framework reframes effort assessment in LLM-assisted development; they do not constitute empirical evaluation or validation of the framework.

## 6 Discussions

### 6.1 Framework-grounded estimation methodology

Although the proposed framework is conceptual in nature, it is intentionally designed to support rigorous quantitative instantiation and empirical analysis. Rather than treating effort as an abstract or purely subjective notion, the framework emphasizes principled links between theoretical constructs and observable interaction- and process-level evidence. This design enables high-level concepts to be translated into measurable variables in a systematic manner, while remaining agnostic to any single operational definition.

As summarized in Table 2, each Hybrid Intelligence Effort dimension is associated with a small set of prioritized candidate operational indicators that capture how effort manifests during human–LLM collaboration. These indicators are derived from artifacts and traces that naturally emerge during LLM-assisted workflows, including prompt–response exchanges, corrective interventions, iteration patterns, code modifications, validation activities, and integration outcomes. Crucially, the indicators in Table 2 are defined at the interaction and process level rather than at the level of specific tools, programming languages, or model internals, enabling consistent measurement across heterogeneous development environments.

**Table 2:** Operational mapping of hybrid intelligence effort dimensions.

**Effort dimension**	**Conceptual meaning**	**Observable indicators and interpretation (prioritized)**
LLM reasoning complexity	The extent to which task intent and constraints are difficult to align with the LLM's generated reasoning, inferred through interaction behavior rather than internal model representations.	• Corrective prompting (primary): Count of explicit human prompts issued to revise, constrain, or redirect the model's output toward task requirements. • Erroneous or inconsistent generations (secondary): Number of model outputs that violate functional expectations, logical consistency, or stated constraints.
Context and information completeness	Degree to which all relevant task, architectural, and domain information is explicitly provided to the LLM at the time of interaction.	• Context-supplementing prompts (primary): Number of additional prompts required to supply missing architectural details, domain knowledge, or codebase context.
Code transformation impact	Breadth and propagation of modifications introduced by the task, particularly across files, modules, or architectural boundaries.	• Affected source artifacts (primary): Total count of files or modules modified as part of task completion. • Cross-boundary modifications (secondary): Evidence of changes propagating across architectural layers or system components. • Post-generation integration or regression failures (Secondary): Number of defects or failures detected during integration or regression validation.
Iterative reasoning cycles	Amount of repeated human–LLM interaction required to converge on outputs that satisfy correctness and quality criteria.	• Prompt–response rounds (primary): Total number of interaction cycles between the developer and the LLM needed to complete the task. • Revision loops (secondary): Count of full generate–assess–correct cycles performed prior to acceptance.
Human oversight effort	Human effort devoted to reviewing, correcting, validating, and integrating LLM-generated artifacts to ensure reliability and accountability.	• Validation and integration findings (primary): Number of defects, inconsistencies, or requirement violations identified during human review and system integration. • Manual artifact revisions (primary): Count of explicit human edits applied to generated outputs to achieve correctness or compliance. • Testing-related activities (secondary): Effort associated with creating, executing, or maintaining validation and regression tests.

The table further distinguishes between *primary* and *secondary* indicators. Primary indicators represent direct and observable manifestations of effort that occur during task execution, such as corrective prompting, interaction iterations, or manual code corrections. Secondary indicators capture downstream or indirect effects, including instability in generated outputs, cross-component propagation, or test-related activities. This prioritization does not impose a hierarchy of importance a priori; instead, it reflects differences in observability and measurement robustness, allowing empirical analysis to determine which indicators exert the strongest influence on realized effort.

The framework deliberately separates conceptual structure from quantitative realization. While Table 2 illustrates one plausible operationalization of each dimension, it does not prescribe fixed metrics, thresholds, or weighting schemes. Instead, it defines a measurement space within which indicators can be selected, refined, or extended based on empirical evidence derived from development logs, execution traces, or controlled experimental observations. This design choice supports adaptability and longitudinal validity: as LLM capabilities, prompting practices, and development workflows evolve, the conceptual dimensions remain stable while their quantitative manifestations may shift. Indicators may therefore be added, modified, or replaced without undermining the theoretical foundation of the framework, allowing it to function as a stable analytical scaffold rather than a fixed or static cost model.

Grounding the conceptual dimensions in operational indicators enables systematic empirical evaluation and comparison with traditional effort estimation approaches. By aligning abstract effort constructs with observable interaction signals, the framework supports quantitative resource estimation, explanatory analysis, and statistical validation, clarifying which interaction dynamics and human oversight activities dominate effort in hybrid human–LLM workflows. Taken together, the proposed framework defines a concrete estimation workflow that can be instantiated empirically without assuming fixed metrics or parametric forms. In practice, framework-based estimation proceeds through the following steps:

1. Capture interaction traces, including prompts, revisions, validation activities, and integration outcomes produced during LLM-assisted task execution.
2. Extract dimension-specific indicators from these traces using the operational mappings defined in Table 2.
3. Normalize indicators across tasks or interaction sessions to enable comparison across heterogeneous development contexts.
4. Learn dimension weights empirically using controlled experiments or observational data and appropriate statistical models.
5. Aggregate weighted dimensions to estimate overall *Hybrid Intelligence Effort* for a given task or development episode.

### 6.2 Implications

#### 6.2.1 Theoretical implications

This work advances software estimation theory by challenging the assumption that effort equals human labor, introducing LLM reasoning as a first-class estimation construct, reframing estimation as an AI–human collaboration problem, and providing a conceptual foundation for future empirical calibration. The introduced framework is positioned as a conceptual successor to traditional effort models for reasoning about software effort in AI-assisted development.

#### 6.2.2 Practical implications

Conceptually, The framework provides a more meaningful estimation basis for LLM-driven workflows. It highlights why story points may become unstable in AI-assisted teams, suggests ways to support more consistent estimation across teams with varying human skill levels, and points toward opportunities for tool-assisted estimation using automated signals from LLM interactions.

Architectural styles and design patterns act as contextual factors that modulate the proposed effort dimensions rather than as determinants of distinct estimation techniques. While prior work has shown that clustering projects by software architecture can improve estimation accuracy in traditional models (e.g., FPA-based approaches Van Hai et al., 2024), the proposed framework captures architectural effects across multiple dimensions: their structural impact is reflected in code transformation scope, while their cognitive and validation overhead is captured through increased LLM reasoning complexity and human oversight effort.

### 6.3 Limitations and future directions

This study is conceptual in nature and has several limitations. First, the framework has not yet been empirically calibrated on large industrial datasets. Second, LLM behavior varies across models and versions, which may affect estimation stability. To account for this variability, future operationalizations of the framework should incorporate a *Model Capability Profile* as a moderating factor influencing LLM reasoning complexity and iterative cycles. This profile may include attributes such as model size, training data composition, fine-tuning specifics, and known behavioral tendencies (e.g., strength in code generation vs. architectural reasoning). By contextualizing effort dimensions within a given model's capabilities, the framework can better adapt to the evolving landscape of LLM technologies and provide more stable estimation foundations across different tooling environments. Additionally, the proposed framework focuses on task-level development effort and does not explicitly model organizational, contractual, or long-term maintenance factors, which may further influence cost dynamics in large-scale or regulated development environments.

Future work should focus on empirical validation, domain-specific calibration, parameterization and scaling of the proposed dimensions, integration with development tools, and longitudinal studies examining estimation accuracy over time. Specifically, each dimension of LLM Cognitive Complexity and Human Oversight Effort should be operationalized with measurable indicators, normalized across tasks and projects, and weighted based on observed contribution to total Hybrid Intelligence Effort, enabling data-driven aggregation and predictive modeling in diverse software development contexts.

## 7 Conclusion

The rise of LLM-assisted software development exposes a fundamental mismatch between modern development practices and traditional effort estimation theory. Human-centric estimation models no longer capture the true drivers of effort when reasoning, coding, and refactoring are partially automated.

This paper provides a conceptual re-examination of software estimation and introduces a unified framework that models effort as a combination of LLM reasoning complexity and human oversight effort. By redefining what is estimated—and how—it lays the groundwork for a new generation of estimation models aligned with AI-augmented software engineering.
