# Supplementary material for: Toward LLM-aware software effort estimation: a conceptual framework
Source: Front Artif Intell. 2026 Mar 23;9:1772418. doi: 10.3389/frai.2026.1772418 (PMC13050940; doi:10.3389/frai.2026.1772418)
Supplement: Supplementary file 1 [file Data_Sheet_1.pdf]

Supplementary Material

SUPPLEMENTARY MATERIAL S1: EXPLORATORY STUDY DESIGN AND OBSERVATIONAL FINDINGS

This supplementary section describes the design and execution of an exploratory empirical study conducted to inform and motivate the proposed LLM-aware software effort estimation framework. The study is not intended as a formal evaluation or empirical validation of the framework. Rather, it serves as an observational analysis used to identify systematic patterns of misalignment between traditional effort estimation techniques and effort incurred in LLM-assisted development workflows.

S1.1 Study Objective

The objective of the exploratory study was to examine whether software development tasks assigned similar traditional effort estimates, expressed in Story Points, exhibit comparable development effort when executed using LLM-assisted workflows. The study specifically aimed to observe how effort distribution changes when core development activities are partially delegated to large language models, and whether Story Point estimates remain stable predictors of effort under these conditions.

S1.2 Task Selection and Dataset

A total of 100 software development tasks were selected from real-world agile datasets that rely on Story Points as the primary estimation mechanism. The selected tasks spanned a broad range of Story Point values and functional characteristics, including algorithmic implementations, feature additions, refactoring tasks, bug fixes, and integration-related modifications.

Task selection intentionally covered tasks historically labeled as low, medium, and high complexity according to Story Point estimates, ensuring representation across the estimation spectrum. The goal was not to construct a statistically balanced benchmark, but to capture realistic variation in task types and estimation practices observed in industrial agile settings.

S1.3 Participants and Execution Protocol

The tasks were independently executed by ten professional software developers with prior experience in both conventional development workflows and LLM-assisted programming. All participants used the same class of contemporary large language models as development assistants.

Developers were instructed to complete 20 tasks using an LLM-assisted workflow in which the LLM was permitted to generate code, propose solutions, and assist with reasoning. Participants retained full responsibility for providing task context and constraints, iteratively refining prompts, reviewing and validating generated outputs, correcting errors, and integrating solutions into the surrounding system context.

Participants were explicitly instructed not to optimize for speed or to artificially constrain interaction with the LLM. Instead, they were asked to work until they judged the solution to be correct, robust, and suitable for deployment. This protocol was designed to surface realistic interaction and oversight effort rather than best-case performance.

S1.4 Observed Dimensions of LLM-Aware Effort

Rather than measuring effort solely through elapsed time or code volume, the study focused on qualitative and process-oriented indicators of effort consistent with the proposed conceptual framework. Observations were recorded regarding the number of prompt–response–refinement cycles required to complete each task, the degree of clarification needed due to incomplete or ambiguous requirements, the scope of code transformations and their propagation across system components, and the intensity of human oversight activities such as testing, debugging, and manual reasoning.

These observations informed the identification of LLM-aware effort dimensions, including LLM reasoning complexity, context and information completeness, iterative reasoning cycles, and human oversight effort. No attempt was made to operationalize these dimensions as numeric variables within the scope of this study.

S1.5 Key Observational Patterns

Across the 100 tasks, several consistent qualitative patterns were observed. Tasks with high Story Point estimates were frequently completed with relatively low LLM-aware effort when they aligned with well-known problem structures or commonly represented patterns. In such cases, LLM-generated solutions required minimal iteration and limited human oversight.

Conversely, tasks with low or moderate Story Point estimates often incurred disproportionately high LLM-aware effort, particularly when they involved legacy codebases, undocumented assumptions, or integration-sensitive changes. These tasks frequently triggered extensive validation, correction, and refinement cycles despite their limited functional scope.

Effort variability within the same Story Point category was substantial, indicating that Story Points failed to cluster tasks by actual effort once LLM interaction dynamics were introduced. In many cases, human oversight effort emerged as the dominant cost driver, even when direct coding effort was substantially reduced through LLM assistance.

S1.6 Implications for Estimation

The exploratory observations suggest that traditional Story Point estimates do not reliably predict effort in LLM-assisted development workflows. Tasks assigned similar Story Point values frequently exhibited markedly different effort profiles once LLM reasoning behavior, interaction dynamics, and validation overhead were taken into account.

These observations support the central argument of the paper that the mismatch between traditional estimation techniques and LLM-assisted development is structural rather than parametric. The findings motivated the reconceptualization of software effort as Hybrid Intelligence Effort, emerging from the interaction between LLM cognitive complexity and human oversight effort, rather than from human coding activity alone.

No quantitative models, correction factors, or empirical performance claims are derived from this study. Instead, the exploratory analysis serves to motivate the need for a new conceptual foundation for software effort estimation in AI-augmented software engineering.
